# Perspectives on Applications of Hierarchical Gene-To-Phenotype (G2P) Maps to Capture Non-stationary Effects of Alleles in Genomic Prediction

**DOI:** 10.3389/fpls.2021.663565

**Published:** 2021-06-04

**Authors:** Owen M. Powell, Kai P. Voss-Fels, David R. Jordan, Graeme Hammer, Mark Cooper

**Affiliations:** ^1^Queensland Alliance for Agriculture and Food Innovation, Centre for Crop Science, The University of Queensland, St Lucia, QLD, Australia; ^2^Queensland Alliance for Agriculture and Food Innovation, Hermitage Research Facility, The University of Queensland, Warwick, QLD, Australia; ^3^ARC Centre of Excellence for Plant Success in Nature and Agriculture, The University of Queensland, St Lucia, QLD, Australia

**Keywords:** multi-trait prediction, non-linear relationships, crop growth models, genetic correlation, non-additive genetic effects, epistasis, pleiotropy, GxE interactions

## Abstract

Genomic prediction of complex traits across environments, breeding cycles, and populations remains a challenge for plant breeding. A potential explanation for this is that underlying non-additive genetic (GxG) and genotype-by-environment (GxE) interactions generate allele substitution effects that are non-stationary across different contexts. Such non-stationary effects of alleles are either ignored or assumed to be implicitly captured by most gene-to-phenotype (G2P) maps used in genomic prediction. The implicit capture of non-stationary effects of alleles requires the G2P map to be re-estimated across different contexts. We discuss the development and application of hierarchical G2P maps that explicitly capture non-stationary effects of alleles and have successfully increased short-term prediction accuracy in plant breeding. These hierarchical G2P maps achieve increases in prediction accuracy by allowing intermediate processes such as other traits and environmental factors and their interactions to contribute to complex trait variation. However, long-term prediction remains a challenge. The plant breeding community should undertake complementary simulation and empirical experiments to interrogate various hierarchical G2P maps that connect GxG and GxE interactions simultaneously. The existing genetic correlation framework can be used to assess the magnitude of non-stationary effects of alleles and the predictive ability of these hierarchical G2P maps in long-term, multi-context genomic predictions of complex traits in plant breeding.

## Introduction

Response to selection in breeding programs relies on predicting the additive genetic merit of new individuals for a target population of environments (Hallauer and Miranda, 1988; Comstock, 1996). Predicting the additive genetic merit of individuals, i.e., breeding values, requires the estimation of allele substitution effects of genetic loci (Falconer and Mackay, 1996). Both functional additive genetic effects and functional non-additive genetic effects, generated by interactions that exist within (dominance) and between (epistasis) genetic loci, contribute to estimates of allele substitution effects (Cheverud and Routman, 1996; Hill et al., 2008; Huang and Mackay, 2016). The contributions of functional additive effects to allele substitution effects are considered stationary as they are not influenced by changes in allele frequencies at genetic loci. However, the contributions of functional non-additive genetic effects (GxG interactions) to allele substitution effects are dependent on the allele frequencies of genetic loci. Therefore, changes in the genetic background can alter the predictions of allele substitution effects. Predictions of allele substitution effects can also change across environments, producing gene-by-environment (GxE) interactions. We refer to the alterations of allele substitution effects, and therefore predictions of the additive genetic merit of individuals in the presence of these interactions as non-stationary effects of alleles. In the most extreme case, allele substitution effects can change sign, i.e., from positive to negative values and *vice versa*, if changes in the value of non-stationary effects exceed the value of stationary effects (Paixão and Barton, 2016; Wientjes et al., 2021). Such sign changes in allele substitution effects change the performance landscape’s optimum and influence the breeding target (Wright, 1963; Messina et al., 2011). Therefore, breeding programs need to accurately predict these non-stationary effects of alleles across different contexts to deliver the highest possible response to selection. Beyond the theoretical considerations, we consider three contexts where the potential for change in sign of allele substitution effects was identified to influence genomic prediction accuracy for commercial maize breeding for the United States corn-belt (Cooper et al., 2014a,b): breeding cycles, populations, and environments. We anticipate these considerations will also be relevant for other plant breeding situations.

Non-stationary effects of alleles decrease the accuracy of genomic predictions across breeding cycles. The accuracy of genomic prediction decreases with an increase in breeding cycles between the training and prediction set (Clark et al., 2012; Pszczola et al., 2012; Daetwyler et al., 2013; Habier et al., 2013). Changes in genetic relationships, linkage disequilibrium, and causal loci’s cosegregation have been identified as important factors (Habier et al., 2013). These factors can impact GxG interactions due to changes in allele frequencies. A practical approach to account for GxG interactions in the decrease in genomic prediction accuracy over breeding cycles is periodic retraining of the genomic prediction equation (Podlich et al., 2004). However, this is costly and may exclude smaller breeding operations. The ability to estimate non-stationary effects of alleles can create opportunities to increase the persistence of prediction accuracy across breeding cycles and widen the application of genomic prediction in plant breeding.

Non-stationary effects of alleles decrease the accuracy of genomic predictions across populations. Genomic prediction across populations is important as the germplasm accessed for breeding applications is often organized in many different populations (Melchinger and Gumber, 1998; Technow et al., 2020; White et al., 2020). Across population prediction often suffers from lower accuracy than prediction across breeding cycles due to more considerable differences in allele frequencies of causal genetic loci (de Roos et al., 2009; Hayes et al., 2009). Along with mutations and redundancy of causal genetic loci, extreme differences in allele frequencies can cause discrepancies in segregation patterns of causal genetic loci between populations, which can cause large differences in allele substitution effects between populations (Rio et al., 2020). Empirical and simulation studies have shown that GxG interactions primarily determine these large changes in allele substitution effects between populations (Duenk et al., 2020; Legarra et al., 2020). Therefore, the ability to accurately capture GxG interactions in genomic prediction will be necessary to effectively utilize diverse germplasm (Tanksley and McCouch, 1997; Jordan et al., 2011; Mace et al., 2013, 2020; Gorjanc et al., 2016; Halewood et al., 2018).

Non-stationary effects of alleles decrease the accuracy of genomic predictions across environments. Genomic prediction across environments has allowed faster identification of stable performing varieties. Most methods that predict performance across environments, including GxE interactions, have been purely statistical (Yates and Cochran, 1938; Finlay and Wilkinson, 1963; Eberhart and Russell, 1966; Piepho, 1997; Burgueño et al., 2012; Crossa, 2012). With implicit knowledge of environmental effects, these methods have been shown to increase prediction accuracy within specific datasets or a well-defined target population of environments. Still, they are sensitive to changes in the target population of environments. Explicit knowledge of environmental effects can make genomic prediction across environments more robust. More recent methods have demonstrated improved prediction accuracy by explicitly including environmental covariates in genomic prediction (Heslot et al., 2014; Jarquín et al., 2014; Costa-Neto et al., 2021; Jarquin et al., 2021). However, all of these methods generate predictions conditional on current environments and therefore represent short-term predictions. Improved long-term predictions of response to selection in plant breeding, including effects of GxE interactions, will require methods to generate predictions of “best-bet” synthetic future environments (Hammer et al., 2020).

Despite the challenge of non-stationary effects of alleles, plant breeding has accurately predicted short-term response to selection to accumulate genetic gain over the long term (Duvick, 2005; Mackay et al., 2011). Short-term predictions of response to selection can mitigate non-stationary effects of alleles by conditioning predictions on current genetic backgrounds and environments. However, with the introduction of genomic prediction (Meuwissen et al., 2001), plant breeding now seeks to re-design breeding programs to further accelerate the pace of varietal development (Bernardo and Yu, 2007; Heffner et al., 2009; Gaynor et al., 2017). The increased speed of selection trajectories of new breeding strategies deploying genomic prediction places a stronger focus on plant breeding programs’ ability to predict long-term response to selection. Long-term predictions of response to selection struggle to mitigate the non-stationary effects of alleles, as predictions conditional on the current genetic background and environment become increasingly uninformative into the future. An illustrative simulation example to explore these concepts is provided in the **Supplementary Information**.

In this perspective, we discuss a few lessons learned from applying hierarchical gene-to-phenotype (G2P) maps in predictive breeding and our view of promising future research directions to realize improvements in the prediction of long-term response to selection in plant breeding.

## Perspective

**FIGURE 1:**
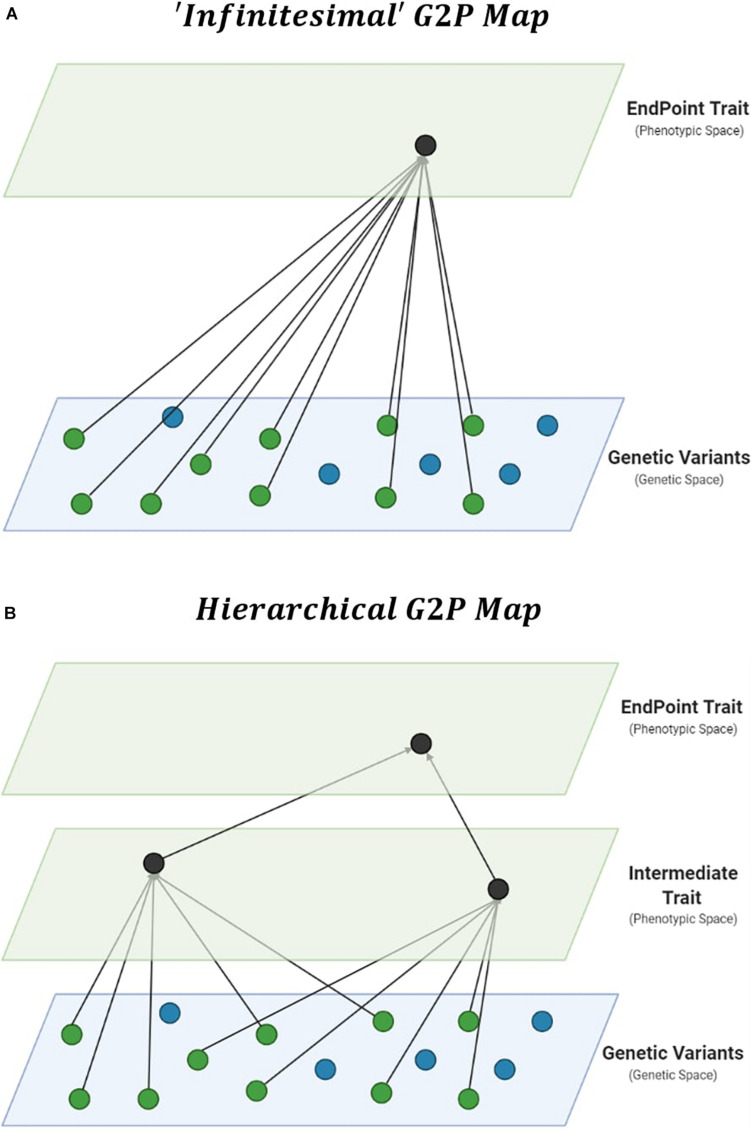
Gene-to-Phenotype (G2P) Maps. **(A)** Representation of an additive infinitesimal G2P map, assuming direct effects of causal genetic variants (green circles) on complex trait phenotypes. **(B)** Representation of an additive hierarchical G2P map, decomposing total effects into direct effects of causal genetic variants on intermediate traits, and phenotypic effects of multiple intermediate traits on complex trait phenotypes.

Improvements in prediction from the specification of interactions require thorough interrogation of the underlying G2P maps of complex traits (Houle et al., 2010; Marjoram et al., 2014). The genetic architecture of traits, which details the number, distribution of effect sizes, and “behavior” of these causal genetic variants, can be viewed as a G2P map. Therefore, the G2P map defines the complete paths from causal genetic variants to the phenotype of complex traits (Waddington, 1957; Burns, 1970; Lewontin, 1974). The dominant G2P map used to investigate the role of interactions in response to selection is a single complex trait underpinned by the infinitesimal model (Robertson, 1960; Carlborg et al., 2006; Hill et al., 2008; Mäki-Tanila and Hill, 2014; Goodnight, 2015; Paixão and Barton, 2016; Wientjes et al., 2021). The infinitesimal model allows breeders to consider complex phenotypes in a single trait context, with underlying genetic variation associated directly with the phenotypic variation of complex traits within a reference population of genotypes (Figure 1A). The infinitesimal model, embedded within the breeders equation (Lush, 1937), has been successful in plant breeding (Hallauer and Miranda, 1988; Comstock, 1996). However, alternative G2P maps have been developed. Here we consider their potential for breeding applications.

Hierarchical G2P maps provide a multi-trait context for investigations into the importance of interactions in genomic prediction. Complex trait phenotypes, such as grain yield, can be viewed as the product of multiple component traits. The hierarchical structure allows intermediate processes (Figure 1B), such as other traits and environmental factors and their interactions, to contribute to complex trait variation (Wright, 1934; Waddington, 1957; Houle et al., 2010; Liu et al., 2019; Cooper et al., 2020a).

In quantitative genetics, hierarchical G2P maps have been developed based on path analysis (Wright, 1934). The specification of intermediate processes in hierarchical G2P maps allows the decomposition of total effects, captured by the infinitesimal G2P map, into path specific direct and indirect effects (Wright, 1934). Lande and Arnold (1983) demonstrated that hierarchical G2P maps could be used to separate direct response to selection from indirect response to selection of multiple correlated traits. Valente et al. (2013) provide an overview of the breeding applications of Structural Equation Models (Gianola and Sorensen, 2004; Pearl, 2012) and highlight their ability to allow prediction across a broader range of livestock and crop management practices than standard multi-trait models without requiring frequent re-estimation of the G2P map. Recently, there has been an increase in the use of Structural Equation Models for prediction and inference in both animal and plant breeding (Tiezzi et al., 2015; Momen et al., 2018; Campbell et al., 2019; Pegolo et al., 2020; Abdalla et al., 2021). However, due to a lack of prior knowledge of the underlying relationships, most studies have used Structural Equation Models to estimate linear relationships between traits. The assumption of linear relationships restricts the range and magnitude of non-stationary effects and, therefore, the frequency of rank changes in additive genetic merit.

**FIGURE 2:**
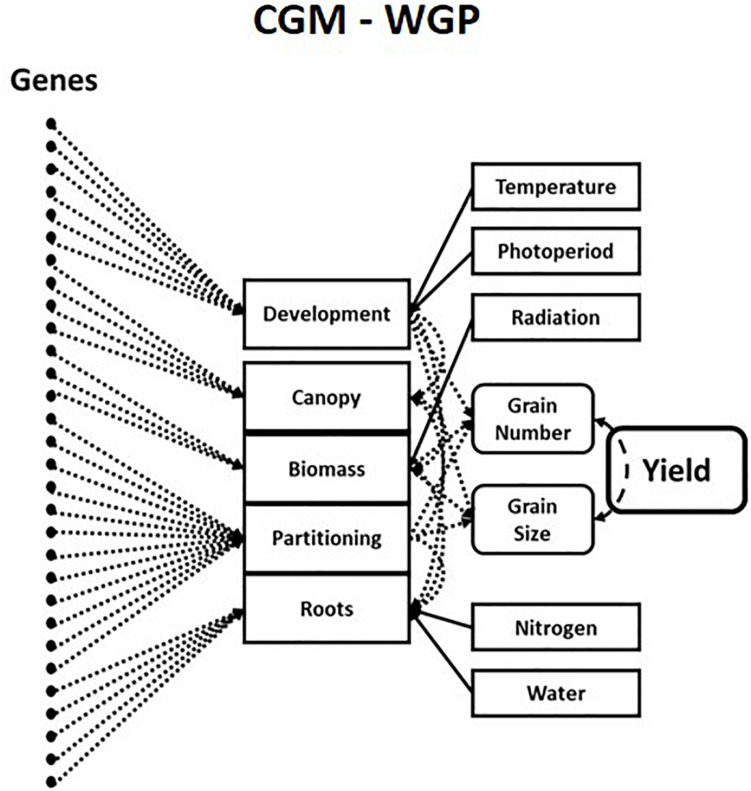
Schematic representation of a hierarchical crop growth model whole genome prediction (CGM-WGP) G2P map. Taken from Figure 2b of Cooper et al. (2020a). Genetic variants are associated with traits or “meta-mechanisms” at lower levels in the crop growth model hierarchy to predict traits at higher levels in the hierarchy.

In plant science, decades of experiments led to the development of hierarchical G2P maps for plant breeding that allow predictions across a wide range of growing conditions (Holzworth et al., 2014; Hammer et al., 2019). Crop Growth Models are hierarchical mechanistic models of plants that simulate trajectories of multiple trait phenotypes over time for the growing season determined by environmental conditions. Crop Growth Models explicitly quantify the relationships, both linear and non-linear, between traits, physiological “meta-mechanisms” and complex trait phenotypes such as grain yield. These “meta-mechanisms” are measurable via high-throughput phenotyping and resulting in robust and stable equations with heritable genotype-dependent parameters (Tardieu et al., 2020). This has allowed Crop Growth Models to be linked to underlying genotypic variation for plant breeding applications (Chapman et al., 2003; Chenu et al., 2009; Messina et al., 2011). More recently, Crop Growth Model – Whole Genome Prediction methods have connected an underlying “infinitesimal” genetic architecture to key components of Crop Growth Models via a hierarchical Bayesian estimation procedure (Figure 2; Technow et al., 2015; Cooper et al., 2016). The inclusion of Crop Growth Models in genomic prediction enables the prediction of trait-trait and trait-environment interactions in the hierarchy’s upper levels, which are directly associated with the estimates of allele substitution effects of genetic parameters for traits in the lower levels of the crop growth model hierarchy. This correction of phenotypes can lead to improved estimates of genetic correlations between traits and increased prediction accuracies across the different contexts discussed above. Crop Growth Model – Whole Genome Prediction methods, and subsequent variations, have been shown to improve short-term predictions of genetic merit in the presence of GxE interactions (Bustos-Korts et al., 2019; Millet et al., 2019; Robert et al., 2020; Toda et al., 2020; Diepenbrock et al., 2021) and genotype-by-environment-by-management interactions in plant breeding. The success of hierarchical G2P maps in capturing non-stationary effects in predictions across diverse environments has seen growth models being revisited in animal breeding (Doeschl-Wilson et al., 2007; Puillet et al., 2016, 2021).

However, the prediction of long-term response to selection remains a significant challenge (Reeve, 2000; Goddard, 2009; Hill, 2017). For example, long-term selection experiments in maize have often produced results not predictable *a priori* or from simulation (Lamkey, 1992; Dudley and Lambert, 2003), such as continued selection response after 100 years (Dudley and Lambert, 2003). Long-term predictions of response to selection, based on the classical versions of the infinitesimal model (Walsh and Lynch, 2018), struggle to accurately predict the non-stationary effects of alleles as information from current genetic backgrounds and environments become increasingly uninformative into the future. A key paper by Paixão and Barton (2016), extending Robertson’s (1960) work with only functional additive effects, has clarified the importance of non-stationary effects of alleles generated by GxG interactions for long-term response to selection. They describe two explicit scenarios: (i) when drift dominates selection, i.e., when the selection pressure at individual functional loci is weak, the initial variance components will determine the increase in response to selection over breeding cycles due to interactions; (ii) when selection dominates drift, i.e., when the selection pressure at individual functional loci is strong, the initial variance components are poor predictors of the response to selection over breeding cycles and details of the G2P map need to be explicitly considered. Therefore, to quantify the importance of non-stationary effects of alleles in predicting long-term response to selection in plant breeding, we should consider two questions:

i. What is the strength of selection operating on the causal loci for traits in breeding programs?
ii. If selection operating on the causal loci is strong, what is the underlying G2P map?

The availability of dense genotype data, sequence data, and advances in phenotyping provide the opportunity to revisit theories about the strength of selection in plant breeding programs. Before the ability to study allelic variation via genotype data, the selection units of breeding programs were breeding values of individuals. It has been shown for complex traits that strong selection at the individual level does not necessarily translate to strong selection at the causal loci (Goddard, 2009; Walsh and Lynch, 2018). However, technologies such as genomic prediction (Meuwissen et al., 2001) are shifting the selection units of breeding programs toward the allele substitution effects of genetic loci. Despite selection still occurring on individuals, genomic selection can distribute selection pressure unevenly across the genome by directing selection pressure to genetic loci with large estimated allele substitution effects (Heidaritabar et al., 2016; Wientjes et al., 2021). Therefore, the use of genomic selection in breeding programs can result in selection dominating drift at specific genetic loci placing greater importance on the G2P map assumed in genomic predictions.

**FIGURE 3:**
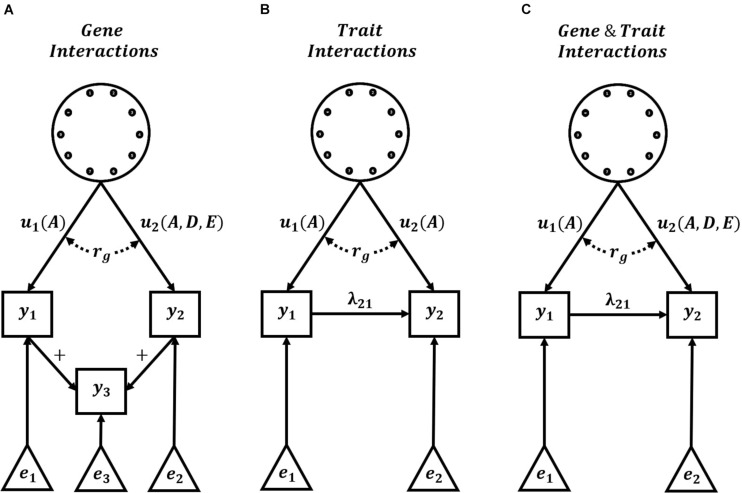
Hierarchical G2P Maps for Plant Breeding. Examples of three multi-trait hierarchical G2P maps with the explicit specification of interactions. Hierarchical G2P maps incorporating knowledge of trait interactions (+, λ) can be used to adjust phenotypes and increase the accuracy of the estimation of gene effects (*u*), gene interactions, and genetic correlations (*r*_*g*_) between traits. Gene effects (*u*) can be directly assigned to trait phenotypes (*y*) or indirectly assigned via linear trait relationships (+) or non-linear trait interactions (λ). *A, D*, and *E* indicate additive, dominance, and epistatic functional genetic effects, respectively. Non-genetic effects of trait phenotypes are represented by *e*. **(A)** Representation of a G2P map with gene interactions and linear relationship between trait phenotypes, **(B)** Representation of current Crop Growth Model – Whole Genome Prediction (CGM -WGP) G2P maps with additive genetic effects and non-linear trait interactions, and **(C)** Representation of potential G2P maps with both gene interactions and non-linear trait interactions.

Complete knowledge of the underlying G2P maps of complex traits is unlikely. However, hierarchical G2P maps with partial knowledge of intermediate processes offer promise for predicting long-term response to selection, given their success in improved short-term predictions of non-stationary effects of alleles. An obstacle in the practical applications of such hierarchical G2P modeling approaches is non-identifiability, also referred to as equifinality or the many-to-one property (Lamsal et al., 2018; Barghi et al., 2020; Henshaw et al., 2020; Kruijer et al., 2020; Tsutsumi-Morita et al., 2021). Effects can be non-identifiable due to unmeasured confounders that generate correlated errors between effects, which results in multiple, equally likely hierarchical G2P maps for experimental data sets. As an example, a multi-trait G2P map involving GxG interactions and the summation of Trait 1 and Trait 2 (Figure 3A) could equally be parameterized as the simplified Crop Growth Model – Whole Genome Prediction G2P map of two traits with purely additive functional genetic effects and non-linear relationships between traits (Figure 3B). Therefore, the level of detail required in hierarchical G2P maps to overcome non-identifiability is still an active research area.

## Future Directions

In recent times, genomic prediction across multiple contexts has received increased focus in breeding (de Roos et al., 2009; Hayes et al., 2009; Windhausen et al., 2012; Gorjanc et al., 2016; Montesinos-López et al., 2019). In a multi-context setting, the genetic correlation naturally provides a measure to quantify predictive accuracy (Falconer, 1952; Robertson, 1959; Bohren et al., 1966). To maximize the benefits of using the genetic correlation framework, plant breeding requires hierarchical G2P maps that include the explicit specification of interactions (Figure 3C). Specification of gene-gene interactions would allow the assessment of changes in the genetic background on GxG interactions and prediction accuracy. Specification of gene-trait and trait-trait interactions would allow the assessment of changes in the environment and agronomic management on GxE interactions and prediction accuracy. Breeding programs are often organized in many different populations or regions to limit these impacts of GxG and GxE interactions, respectively, while assuming a single performance optimum and single breeding target. However, GxG or GxE interactions can generate a performance landscape with multiple optima (Wright, 1963; Cooper et al., 2005; Messina et al., 2011; Technow et al., 2020). Prior specification of this multiple optima landscape, via hierarchical G2P maps, would allow more comprehensive explorations of the impact of such interactions on the long-term response to selection of plant breeding programs.

Complementary simulation and empirical studies can interrogate the changes of genetic correlations across contexts to quantify the relative magnitude of GxG and GxE interactions and measure their impact on genomic prediction. Recent research, primarily from animal breeding, has renewed the focus on this framework (Wientjes et al., 2015; Dai et al., 2020; Duenk et al., 2020; Legarra et al., 2020). The common theme has been using the genetic correlation to assess likely magnitudes of GxG interactions underpinning complex traits. Duenk et al. (2020) used simulations to show that realistic levels of dominance alone could not drive the genetic correlation between two populations below 0.8, but realistic levels of epistasis could drive the genetic correlation as low as 0.45. Legarra et al. (2020) used two regularly intermated populations with similar allele frequencies and an expectation of minimal GxG interactions to speculate on the role of GxE in low across population predictions. They also suggested a genetic correlation threshold of 0.6, below which populations should be classed as distinct. However, these recent animal breeding studies overlooked the inclusion of GxE interaction scenarios. GxE interaction scenarios are of high relevance to plant breeding which regularly predict across a diverse set of target population of environments. Plant breeding is in a prime position to use results from evolutionary genetics (de Villemereuil et al., 2016), multi-environment trial analyses (Piepho, 1997; van Eeuwijk et al., 2005; Malosetti et al., 2013), and Crop Growth Models (Jones et al., 2003; Hammer et al., 2010; Messina et al., 2011; Holzworth et al., 2014) to assess the impact of GxE interactions on genetic correlations and determine their influence on breeding programs designed to utilize genomic prediction. Therefore, we propose that the plant breeding community undertake complementary simulation and empirical studies to quantify the relative magnitude of GxG and GxE interactions across relevant environmental and population contexts to quantify their impact on genomic prediction.

The dominant crop improvement procedure of today is a sequential operation. Breeding programs first develop new varieties with a limited sampling of the full range of farmers’ agronomic possibilities. Within this first step, plant breeding programs simultaneously perform population improvement to improve the additive genetic merit of breeding germplasm and product development, to identify new varieties with the highest total genotypic merit (Messina et al., 2011; Powell et al., 2020; Technow et al., 2020; Werner et al., 2020). Then agronomic research programs follow, focusing on developing and optimizing crop management strategies for the handful of new varieties. Hierarchical G2P maps can connect the objectives of plant breeding and quantitative genetics with those of crop agronomy (Figure 3; Cooper et al., 2020a,b). The explicit connections between gene and multiple trait levels, embedded in hierarchical G2P maps, can be perturbed experimentally (empirical and simulation) to quantify the impact of agronomic management interventions and changes in the environment. The effects of the perturbations can be investigated to determine how they propagate through the hierarchical G2P map and update estimates of allele effects at both the gene and trait levels. *Ex-ante* predictions of perturbations at the gene level could be used to guide improved prediction of “synthetic” varieties developed through novel gene-editing techniques. *Ex-ante* predictions of perturbations at the trait level could improve the efficiency of breeding new varieties adapted for alternative farming systems and future climate scenarios (Hammer et al., 2020). At the same time, predictions can be extracted from each level of the hierarchical G2P map, allowing the decomposition of individual performance into additive genetic, total genetic, and phenotypic merit. Decomposition of path-specific values in hierarchical G2P maps has been demonstrated in evolutionary and quantitative genetics (Lande and Arnold, 1983; Gianola and Sorensen, 2004; Valente et al., 2010, 2013; Henshaw et al., 2020; Janeiro et al., 2020; Pegolo et al., 2020). Therefore, the ability to exploit different sources of improved crop performance under a single prediction framework could improve crop improvement pipelines’ accuracy and flexibility to navigate performance landscapes for current and future environments (Messina et al., 2011, 2020; Technow et al., 2020).

## Conclusion

Current genomic prediction methods struggle to predict the non-stationary effects of alleles as the genetic background (breeding cycles and populations) and the environment changes. These non-stationary effects of alleles are determined by interactions between genetic loci, traits, and the environment. Non-stationary effects of alleles result in low prediction accuracy across breeding cycles, populations and environments. As discussed above, the development of hierarchical G2P maps has been shown to improve the genomic prediction of non-stationary effects of alleles across breeding cycles and environments. The simultaneous specification of GxG and GxE interactions in hierarchical G2P maps may help to more thoroughly explore the impact of non-stationary effects of alleles on the long-term response to selection of plant breeding programs.

## Data Availability Statement

The original contributions presented in the study are included in the article/Supplementary Material, further inquiries can be directed to the corresponding author/s.

## Author Contributions

OP and MC conceived and designed the perspective. OP wrote the first manuscript draft and developed the supporting simulations. MC, KV-F, DJ, and GH helped to refine the manuscript. All authors read and approved the final manuscript.

## Conflict of Interest

The authors declare that the research was conducted in the absence of any commercial or financial relationships that could be construed as a potential conflict of interest.

## Supplementary Information

A walkthrough of a simulation, data files, and scripts demonstrating non-stationary effects of alleles over breeding cycles can be accessed at https://powellow.github.io/Interactions_In_Breeding/.
